# Inequity of healthcare access and use and catastrophic health spending in slum communities: a retrospective, cross-sectional survey in four countries

**DOI:** 10.1136/bmjgh-2021-007265

**Published:** 2021-11-29

**Authors:** The Improving Health in Slums Collaborative

**Keywords:** health economics, health systems evaluation, cross-sectional survey, health policy

## Abstract

**Introduction:**

Tracking the progress of universal health coverage (UHC) is typically at a country level. However, country-averages may mask significant small-scale variation in indicators of access and use, which would have important implications for policy choice to achieve UHC.

**Methods:**

We conducted a retrospective cross-sectional household and individual-level survey in seven slum sites across Nigeria, Kenya, Bangladesh and Pakistan. We estimated the adjusted association between household capacity to pay and report healthcare need, use and spending. Catastrophic health expenditure was estimated by five different methods.

**Results:**

We surveyed 7002 households and 6856 adults. Gini coefficients were wide, ranging from 0.32 to 0.48 across the seven sites. The total spend of the top 10% of households was 4–47 times more per month than the bottom 10%. Households with the highest budgets were: more likely to report needing care (highest vs lowest third of distribution of budgets: +1 to +31 percentage points (pp) across sites), to spend more on healthcare (2.0 to 6.4 times higher), have more inpatient and outpatient visits per year in five sites (1.0 to 3.0 times more frequently), spend more on drugs per visit (1.1 to 2.2 times higher) and were more likely to consult with a doctor (1.0 to 2.4 times higher odds). Better-off households were generally more likely to experience catastrophic health expenditure when calculated according to four methods (−1 to +12 pp), but much less likely using a normative method (−60 to −80 pp).

**Conclusions:**

Slums have a very high degree of inequality of household budget that translates into inequities in the access to and use of healthcare. Evaluation of UHC and healthcare access interventions targeting these areas should consider distributional effects, although the standard measures may be unreliable.

**Key questions**

**What is already known?**

- We previously identified 20 narrative reviews and 128 cohort studies describing epidemiology determinants of health, and healthcare use in slum settings through a systematic search of MEDLINE and Embase in 2016, which showed that people living in slums encountered many barriers in accessing health services.
- While previous studies describe differences in provision of health services, for example, a preponderance of private providers in India and public facilities in Ethiopia and Kenya, they do not provide systematic detail or analysis of factors determining use of facilities including income, nor do they report distributional differences and inequalities in these neighbourhoods.

**Key questions**

**What are the new findings?**

- We find evidence of significant inequalities in household expenditure across all the seven sites and that these inequalities in spending translate into inequalities in healthcare use with the best-off households visiting doctors and nurses up to three times as frequently as the worst-off.
- We also find evidence of high rates of catastrophic health expenditure, however, better-off households were more likely to experience catastrophic health expenditure according to four out of the five methods we used, suggesting that these measures were relatively poor at identifying ‘affordability’.

**What do the new findings imply?**

- Broad neighbourhood-level policies to improve healthcare access may have the effect of exacerbating healthcare inequalities without specific support for the worst-off households.
- The distributional consequences of interventions should be routinely included in evaluations of healthcare access interventions to identify whether the worst-off households are benefitting.

## Introduction

The aim of universal health coverage (UHC) is to ensure that everyone gets the care they need regardless of their ability to pay, and that no-one suffers undue financial hardship as a result of seeking healthcare.^1^ There has been a growth in the commitment worldwide to achieving UHC and it was adopted as one of the United Nations’ Sustainable Development Goals (SDGs; indicator 3.8.2). Countries are responding by designing programmes to migrate towards UHC.

It is widely agreed that supply-side healthcare reforms should be pro-poor, including measures like elimination of user fees,^2^ introduction of public or social insurance schemes,^3^ or increased local public provision of services.^4^ However, identifying poor people specifically has limitations: it is costly, inaccurate and risks ‘adverse selection’. As a result many programmes target ‘people in poor neighbourhoods’^5^ instead.

An archetypal ‘poor neighbourhood’ is a slum or informal settlement. Slums are often the target of localised healthcare interventions given the definitional lack of services and access to care in these neighbourhoods^6 7^; indeed, the UN’s SDGs identifies addressing ‘the plight of slums’ as a global priority. UHC is a statement though about individual level rights and protections and so targeting and monitoring. However, targeting programmes at a slum-level only makes sense if the people living in slums are homogeneously poor relative to the country at large and if the response to intervention would be to improve healthcare access for all residents.

There are several indicators widely used to monitor UHC progress, including examining the association between income and care use, adjusting for proxies for healthcare need, and examining catastrophic health expenditure (CHE) rates. CHE is most commonly measured as healthcare expenditures exceeding 10% or 25% of a household’s total budget, measured either as income or consumption expenditure.^8 9^ At a country level, a low incidence of CHE may simply reflect low service coverage.^4^ However, at a more granular level (within country or community) better-off households may spend more on healthcare both in absolute and proportionate terms when faced with the same service coverage, so better-off households may also be more likely to be classified as experiencing CHE even though the expenditure was affordable for them.^8^ Thus, a slum may appear to be getting worse-off by some measures of CHE when in fact their material circumstances are improving, or better-off households use more healthcare, and service coverage remains unchanged with significant gaps for the poorest. Alternative methods of calculating CHE have been proposed to try to combat this effect, but the results can vary widely, and have seldom been examined at community levels. The only previous slum-specific study on CHE showed that the proportion of households experiencing CHE in two Kenyan slums varied between 2% and 28% depending on the method used^10^

Recent reports have reported generally positive progress internationally on UHC using these indicators at the country level.^4 11 12^ However, there exists little previous evidence on the values one might expect of indicators of inequitable access and financial hardship within a slum or similar community, nor whether the most commonly used measures can provide a useful insight into the performance of UHC programmes at this level. Thus, there remains questions about healthcare access in poor communities and how to monitor it.

In this study, we examine inequalities of healthcare need, access, use and expenditure within slums. We first consider income and household budget inequalities. Second, we estimate the association between these inequalities and reported healthcare need, access and use. And third, we estimate five indicators of CHE for each household and explore how they relate to each other and within-slum inequalities. Our objective is to provide both a reference for future evaluations of access-promoting interventions, and to consider the usefulness of such measures within these communities. We study seven slums across four countries with significant populations of urban poor residents: Nigeria, Kenya, Pakistan and Bangladesh.

## Methods

### Data collection

We conducted a retrospective, cross-sectional household and individual survey across seven slum sites in Nigeria, Kenya, Bangladesh and Pakistan. We refer to these sites pseudonymously as NG1 and NG2 (Ibadan, Nigeria), NG3 (Lagos, Nigeria), KE1 and KE2 (Nairobi, Kenya), PK1 (Karachi, Pakistan) and BD1 (Dhaka, Bangladesh). Full details of the survey methodology are published elsewhere.^13^

All sites were mapped using remotely-sensed data, which was then ground-truthed using a participatory mapping process, and any errors corrected. All resident households were listed in this stage to form a sampling frame, from which we drew a spatially-regulated^14^ random sample of 1200 households from each site, with the goal of achieving a sample of 1000 households. Field workers made up to three attempts to visit each sampled household. A member of each consenting household (typically the head of household) completed a survey on household-level information including a roster with demographic and socioeconomic information, and household income and spending across various categories (including rent, food, water, electricity and healthcare). An adult and a child under 12 were randomly sampled from the household roster to complete individual-level surveys. We sampled adult women at a ratio of 2:1 with respect to adult men as we hypothesised that women would use healthcare more frequently than men. The individual-level survey collected information on a range of healthcare need, use and spending. Data quality control procedures were used in the survey process including spot checks and sit-ins by field supervisors and computer checks of submitted data with any erroneous entries sent back to the field for correction. Surveys were translated using iterative process involving forward and independent backward translations.

### Patient and public involvement

Mapping of the study sites, identification of healthcare facilities and enumeration of resident households was conducted using a participatory process involving local residents. Healthcare facility managers and owners were consulted about identification of their facilities. The public were not involved in the design of the survey questionnaires, however feedback was sought from residents in a pilot survey in all sites to assess the time burden of participating. Patient and public focus groups were established to present the findings, receive feedback and provide contextualising interpretation of the results.

### Statistical analysis

#### Household budget

For every household we calculated its *total budget*, which we defined as the total monthly household consumption expenditure. We also calculated the household budget per equivalent person. ‘Equivalent persons’ were calculated using the square root of the number of household members to account for the economies of scale of living in larger households.^15 16^ Households were divided into thirds within each site based on their position in the distribution of household budget per equivalent person (bottom, middle and top) for the adjusted analyses of association of household budget with healthcare need, use and spending.

#### Budget inequalities

To summarise household budget inequality within each site we calculated three measures: (i) the Gini coefficient for the total household budget; (ii) the ratio of the 90th to 10th percentiles of household budget (‘90/10 ratio’); and (iii) the ratio of the 90th to 10th percentiles of household budget per equivalent person.

#### Inequalities of need and use

Individuals were asked if they had needed healthcare in the previous 12 months and if so whether they had received healthcare. We estimated absolute risk differences in the proportion of respondents reporting healthcare need and if they had received it by thirds of household budget per equivalent person adjusted for age, age squared, sex, highest level of education completed and whether the respondent had a long-term health condition.

At the household level we extracted *healthcare* spending in the previous month and estimated a log-linear model (log healthcare expenditure against household budget third) adjusted for number and age of household members. At the individual-level we also extracted: number of healthcare visits in the previous year not involving an overnight stay, number of visits with an overnight stay, whether a visit without an overnight stay was to a doctor and the amount spent on a visit without an overnight stay on consultancy fees, drugs and tests. For each of the individual-level outcomes we estimated a regression model separately by site (Poisson for count data for an incidence rate ratio, binomial-logistic for binary for an OR and log-linear for continuous for an elasticity) of each outcome on their household’s budget third, adjusted for the individual’s age, age squared, sex, household size, highest education level achieved and whether they had any long-term conditions.

#### Catastrophic health expenditure

Following Cylus *et al*^8^ we calculated five measures of CHE for each study site. Each measure was defined based on the ratio of healthcare spending to the household’s capacity to pay. In addition to the household’s total budget described above, the other measures of household ‘capacity to pay’ were:

- *Actual food spending*: The *total budget* minus the household’s actual monthly spending on food.
- *Partially normative food*: The *total budget* minus an amount representing subsistence food spending, except for households already below the subsistence amount for whom actual food spending was subtracted. Subsistence food spending was determined as the average food spending per equivalent person among households whose food share of total spending was between the 45th and 55th percentile.
- *Normative spending on food, rent and utilities*. The *total budget* minus an amount representing subsistence spending on food, rent and utilities, which was defined as the mean spending on these items per equivalent person for households that were between the 25 and 35 percentiles of total budget.

The five CHE definitions were:

- Methods 1 and 2: 10% and 25% of total budget, respectively (used by WHO’s Global Health Observatory, World Bank, and others);
- Method 3: 40% of actual food spending (used by Pan American Health Organisation and World Bank);
- Method 4: 40% of partially normative food spending (used by WHO)
- Method 5: 40% of normative spending on food, rent and utilities (used by WHO’s Regional Office for Europe).

We examined the proportion of households identified as experiencing CHE for each method and we determined their agreement as the proportion of households classified in the same way by each method. Finally, we estimated absolute risk differences in the proportion of households with CHE by thirds of household budget per equivalent person adjusted for the number and age of household members.

## Results

Table 1 reports summary statistics of the study populations estimated from the samples. Overall, we interviewed 7002 households and 6857 adults. The seven sites were broadly comparable in demographic characteristics, although the Nigerian sites had moderately higher educational levels than elsewhere, and employment rates were lower in sites KE1 and PK1 (55% and 46%, respectively) than elsewhere (61%–70%).

**Table 1:** Summary statistics

		Nigeria			Kenya		Pakistan	Bangladesh
		NG1	NG2	NG3	KE1	KE2	PK1	BD1
N	Households	1256	844	812	1015	1089	957	1029
	Individuals (all)	4794	2936	3440	3878	2724	6077	4323
	Individuals (healthcare survey, adults)	1228	838	802	1004	1085	919	981
Age (%)	Under 5	11 (10, 12)	9 (8, 10)	10 (9, 11)	13 (12, 14)	12 (10, 13)	11 (10, 12)	11 (10, 12)
	5–19	34 (32, 35)	31 (29, 33)	33 (32, 35)	37 (35, 38)	26 (24, 28)	30 (29, 31)	32 (31, 33)
	20–44	36 (35, 37)	35 (33, 37)	39 (37, 40)	37 (35, 38)	51 (50, 53)	41 (40, 42)	45 (43, 46)
	45–64	14 (13, 15)	17 (15, 18)	15 (14, 16)	11 (10, 12)	10 (9, 12)	15 (14, 16)	10 (9, 11)
	65 and over	5 (4, 6)	8 (7, 9)	3 (3, 4)	3 (2, 3)	1 (0, 1)	4 (3, 4)	2 (2, 3)
Sex (%)	Male	50 (48, 51)	50 (48, 51)	50 (49, 52)	50 (49, 52)	55 (53, 56)	51 (50, 53)	53 (51, 54)
Education (completed) (%)	Primary	13	16	17	25	23	13	31
	Secondary	24	31	34	13	30	9	4
	Tertiary	11	6	20	5	6	0	4
Currently employed (adults) (%)		61 (60, 63)	70 (68, 71)	69 (68, 71)	55 (53, 56)	68 (66, 70)	46 (44, 47)	70 (68, 71)
Long term health conditions (%)		8 (7, 8)	13 (12, 14)	5 (5, 6)	12 (11, 13)	10 (9, 11)	15 (15, 16)	12 (11, 13)
Healthcare spending (monthly Int$; median (IQR))	Total	0(0, 13)	0 (0, 13)	2 (0, 25)	1 (0, 9)	2 (0, 8)	16 (0, 87)	24 (9, 61)
	Per person	0 (0, 4)	0 (0, 4)	0 (0, 6)	0 (0, 3)	1 (0, 3)	3 (0, 15)	7 (3, 18)
Capacity to pay(monthly Int$; median (IQR))	Total budget	254 (105, 428)	257 (108, 432)	467 (291, 736)	194 (131, 309)	211 (147, 305)	1008 (683, 1,451)	377 (266, 538)
	Actual food spending	101 (48, 196)	113 (56, 209)	233 (139, 399)	90 (48, 160)	93 (64, 156)	505 (315, 814)	170 (108, 300)
	Partially normative food	142 (60, 282)	147 (65, 257)	272 (148, 494)	114 (61, 201)	111 (72, 191)	581 (351, 959)	205 (126, 354)
	Normative food, rent, utilities	−25 (−168, 122)	−5 (−142, 148)	46 (−133, 283)	56 (−4, 154)	26 (−34, 106)	86 (−177, 495)	111 (23, 256)
Measures of inequality of household budgets	Gini coefficient	0.48	0.48	0.38	0.37	0.34	0.34	0.32
	90/10 ratio household budget	46	39	7	6	4	5	4
	90/10 ratio household budget per equivalent person	49	34	6	5	4	4	3
Catastrophic health expenditure (%)	Total budget (10%)	12 (10, 14)	12 (10, 14)	12 (10, 15)	14 (12, 16)	9 (7, 10)	21 (18, 23)	38 (35, 41)
	Total budget (25%)	5 (4, 6)	6 (5, 8)	4 (2, 5)	4 (3, 5)	2 (1, 3)	6 (5, 8)	15 (13, 17)
	Actual food spending (40%)	8 (6, 9)	7 (5, 9)	4 (3, 6)	6 (4, 7)	2 (1, 3)	8 (7, 10)	18 (16, 20)
	Partially normative food (40%)	5 (4, 6)	5 (4, 7)	3 (2, 5)	5 (4, 6)	2 (1, 3)	7 (5, 8)	15 (13, 18)
	Normative food, rent, utilities (40%)	30 (28, 33)	31 (27, 34)	22 (19, 25)	25 (22, 27)	30 (28, 33)	29 (26, 32)	33 (30, 36)
Healthcare need and use (individual-level)	Needed care last 12 months (%)	59 (57, 60)	56 (54, 57)	51 (49, 52)	87 (86, 88)	87 (86, 89)	64 (63, 65)	97 (96, 97)
	Received care when needed (%)	99 (99, 99)	98 (98, 99)	97 (97, 98)	96 (96, 97)	98 (98, 99)	96 (96, 97)	97 (96, 97)
	Annual number of visits without overnight stay	1.4 (1.3, 1.5)	1.4 (1.3, 1.6)	1.1 (1.0, 1.2)	2.8 (2.6, 2.9)	2.7 (2.5, 2.8)	1.9 (1.8, 2.0)	5.2 (5.0, 5.4)
	Annual number of visits with overnight stay	0.1 (0.1, 0.2)	0.1 (0.1, 0.2)	0.1 (0.1, 0.1)	0.1 (0.1, 0.1)	0.0 (0.0, 0.1)	0.1 (0.0, 0.1)	0.1 (0.1, 0.1)
	Visits to a doctor (%)	19 (17, 21)	18 (16, 20)	17 (14, 19)	38 (36, 41)	38 (35, 40)	52 (50, 55)	23 (21, 25)
	Consult fees per visit (Int$; mean (95% CI))	1.27 (1.00 to 1.54)	1.19 (0.78 to 1.60)	1.12 (0.83 to 1.42)	0.74 (0.56 to 0.92)	0.42 (0.33 to 0.52)	2.36 (2.14 to 2.59)	1.56 (1.36 to 1.75)
	Drugs spend per visit (Int$; mean (95% CI))	8.08 (7.28 to 8.89)	6.97 (6.05 to 7.90)	7.66 (6.63 to 8.69)	3.18 (2.83 to 3.53)	5.24 (4.79 to 5.70)	9.72 (8.66 to 10.78)	11.03 (10.30 to 11.76)
	Tests spend per visit (Int$; mean (95% CI))	1.33 (1.09 to 1.56)	1.07 (0.81 to 1.34)	1.25 (0.94 to 1.55)	0.70 (0.50 to 0.90)	1.04 (0.84 to 1.24)	1.74 (1.08 to 2.39)	1.40 (1.09 to 1.71)

BD1, Dhaka, Bangladesh; Int$, International dollars; KE1 and KE2, Nairobi, Kenya; NG3, Lagos, Nigeria; NG1 and NG2, Ibadan, Nigeria; PK1, Karachi, Pakistan.

### Household budget inequality

**Figure 1:**
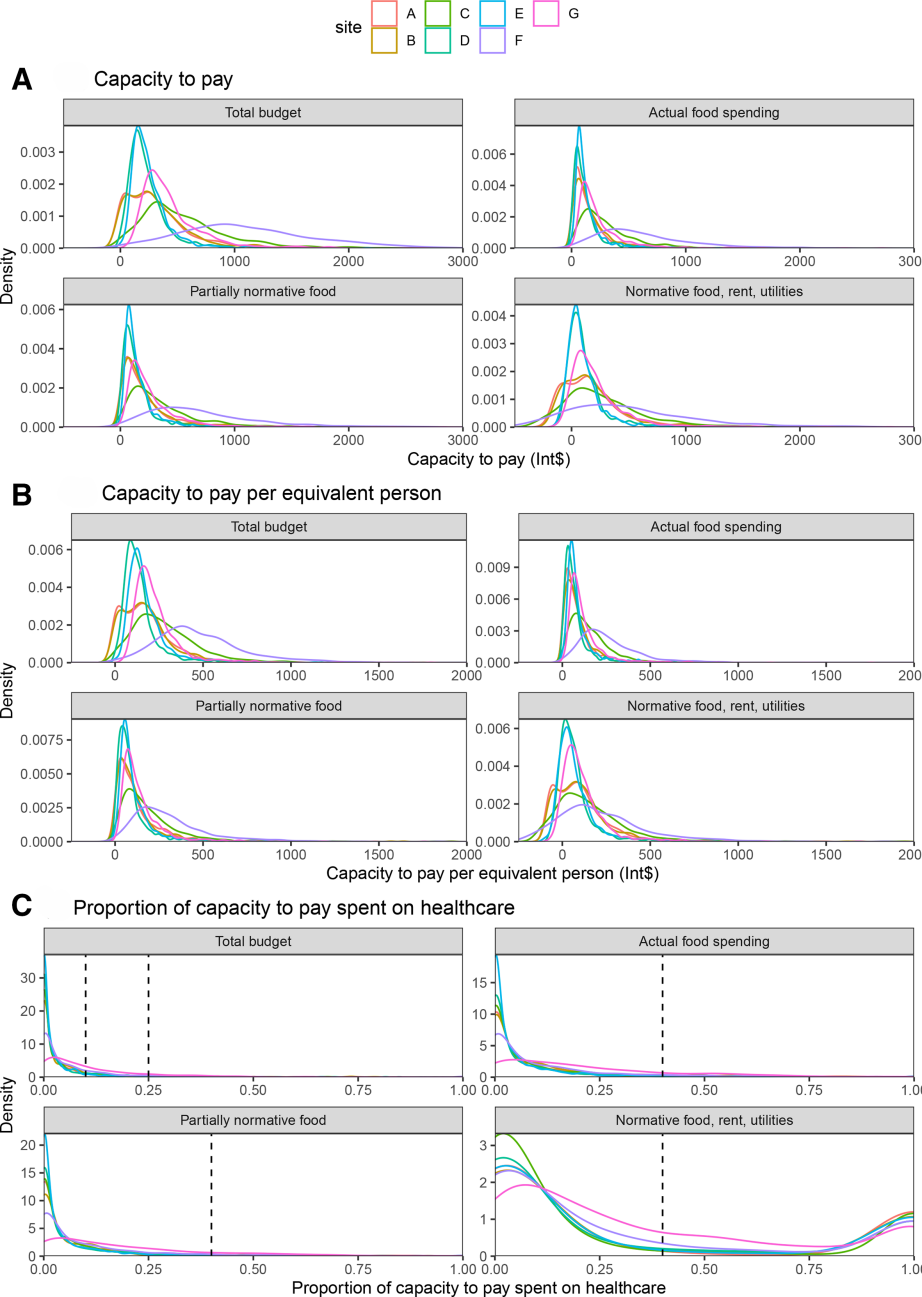
(A) Density plot of the different measures of capacity to pay for each site. (B) Density plot of the different measures of capacity to pay divided by the number of equivalent persons in each household. (C) Density plot of the proportion of capacity to pay spent on healthcare with vertical lines indicating catastrophic health expenditure thresholds. Int$, International dollars

Figure 1A, B show the different measures of capacity to pay. Median household total budget was highest in Pakistan (International dollars (Int$)1008 vs Int$194–467 elsewhere). While households had approximately double the number of members in PK1 compared with other sites, it still had the highest median total budget per equivalent person. Gini coefficients were wide ranging with respect to total household budgets: from 0.32 in BD1 to 0.48 in NG1 and NG2. The 90/10 ratios for sites NG1 and NG2 were 46 and 39, indicating the top 10% of households spent 46 and 39 times more per month than the bottom 10%, respectively, in these sites. These values were much greater than the other sites, which nevertheless ranged from 4 to 7.

### Inequalities of need and use of health services

#### Healthcare need

In Kenya and Bangladesh, the vast majority of respondents reported requiring healthcare at any point in the previous year (87% in Kenya and 97% in Bangladesh) whereas in Nigeria and Pakistan the proportions were lower (51%–64%) (table 1). However, in all countries 96% or more reported receiving care when they perceived a need for it. Table 2 reports adjusted risk differences for the probability of reporting needing care by third of the distribution of total budget per equivalent person. Households with the highest budgets were more likely to report needing care (highest vs lowest third: 0.6 to 31.0 percentage points higher), except for in Bangladesh where, as stated, almost all reported needing care. The differences were greatest in sites NG1 and NG2. There was little evidence that children were more likely to report needing care in the best-off households (eg, highest to lowest thirds: −16.0 to 4.9 percentage points). We did not estimate models for receiving care if needed as there was little to no variation in the outcome, given that 98% or more reported receiving care when a health need was perceived.

**Table 2:** Adjusted absolute risk differences (percentage point) in the probability of reporting a need for healthcare in the previous 12 months by tertile of household consumption expenditure per equivalent person

Adults	NG1	NG2	NG3	KE1	KE2	PK1	BD1
Bottom	Ref.	Ref.	Ref.	Ref.	Ref.	Ref.	Ref.
Middle	22.6 (16.3, 28.9)	7.0 (−0.5, 14.4)	7.3 (−0.4, 15.0)	10.6 (4.4, 16.8)	−1.8 (−7.0, 3.5)	7.0 (−1.3, 14.1)	−0.1 (−2.5, 2.3)
Top	31.0 (16.3, 28.9)	14.1 (6.6, 21.6)	7.3 (−0.5, 15.0)	11.0 (4.8, 17.3)	2.2 (−3.2, 7.5)	4.7 (−2.4, 11.9)	0.6 (−1.9, 3.0)
**Children**	**NG1**	**NG2**	**NG3**	**KE1**	**KE2**	**PK1**	**BD1**
Bottom	Ref.	Ref.	Ref.	Ref.	Ref.	Ref.	Ref.
Middle	−3.7 (−19.5, 12.3)	5.6 (−17.0, 28.1)	−15.4 (−35.7, 5.0)	0.9 (−5.0, 6.9)	7.1 (−0.2, 14.4)	5.7 (−2.8, 14.2)	4.2 (0.8, 7.5)
Top	−16.0 (−32.2, 0.3)	−4.0 (−26.8, 18.8)	−1.7 (−24.9, 21.4)	4.6 (−1.9, 11.0)	4.1 (−4.5, 12.7)	4.9 (−3.7, 13.6)	1.5 (−1.9, 4.9)

BD1, Dhaka, Bangladesh; KE1 and KE2, Nairobi, Kenya; NG3, Lagos, Nigeria; NG1 and NG2, Ibadan, Nigeria; PK1, Karachi, Pakistan.

#### Overall health spending

**Figure 2:**
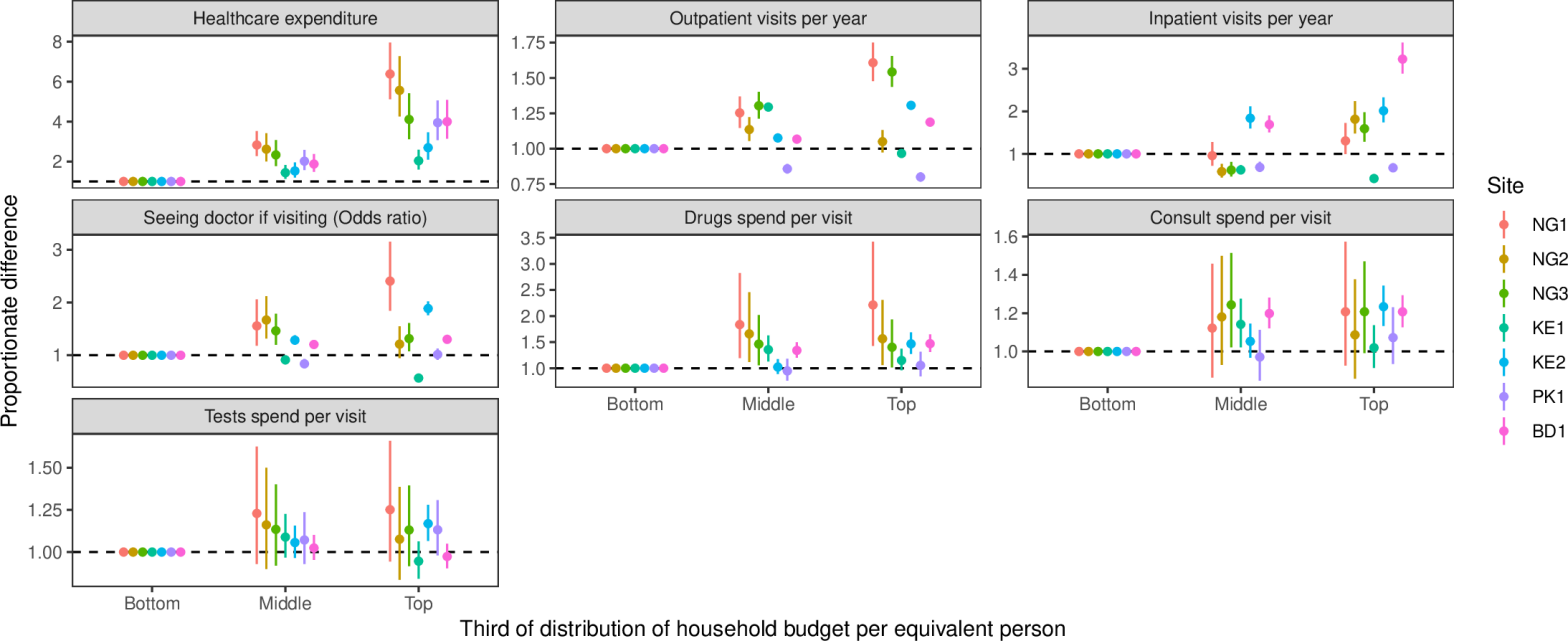
Adjusted proportionate difference in healthcare use and expenditure, overall and per visit, between thirds of the distribution of household budget per equivalent person (‘Bottom’ is reference category). BD1, Dhaka, Bangladesh; KE1 and KE2, Nairobi, Kenya; NG1 and NG2, Ibadan, Nigeria; NG3, Lagos, Nigeria; PK1, Karachi, Pakistan.

Better-off households spent more both absolutely and proportionately on healthcare in all sites after adjusting for socio-demographic characteristics. Figure 2 shows the estimated proportionate difference in healthcare spending between thirds of the distribution of household budget per equivalent person adjusted for age, sex and other characteristics. The top third of households spent 2.0 to 6.4 times more than those in the bottom third.

#### Type of healthcare use

Adjusted inpatient and outpatient visit rates were higher in Nigeria, Pakistan and Bangladesh for better-off households (top vs bottom third: 1.0 to 3.0 times more frequently), and patients in these locations were more likely to see a doctor when they sought care (1.0 to 2.4 times higher odds). The mean spending on drugs per visit was higher for better-off households in all sites (1.1 to 2.2 times higher), however there was little evidence of a difference in spending on consultation fees and tests per visit.

### Catastrophic health expenditure

#### Comparison of methods

Table 1 and figure 1A, B report the capacities to pay for healthcare within each site. Site PK1 had the highest capacity to pay according to all measures, except for normative spending on food, rent and utilities (method 5), in which BD1 had the highest capacity to pay. Sites NG1 and NG2 had a negative median capacity to pay using the normative method (method 5) (Int$−25 and Int$−5). The African sites were all lower than PK1 and BD1 for all measures.

Figure 1C shows the distribution of the proportion of each capacity to pay spent on healthcare within each site and table 1 reports the proportions of households classified as experiencing CHE using each of the five methods. For methods based on household budget, actual food spending and partially normative spending (methods 1–4), between 2% and 18% of households had CHE. Bangladesh was also the highest on each of these measures (15%–18% versus 2%–8% elsewhere). However, for the normative spending method (method 5), between 24% and 33% of all households had CHE and Bangladesh had comparable rates to the other sites. Table 1 also shows the reported mean spending per outpatient visit (including clinic, hospitals and pharmacies); the costs in Bangladesh were comparable to other sites, however the visit rate was about twice as frequent as elsewhere (5.2 *vs* 1.1 to 2.8 visits per person-year elsewhere). Table 3 shows the agreement between the different methods. Methods 1–4 classify between 88% and 98% of the households in the same way, however the agreement between method 5 and methods 1–4 was 78% to 82% so that approximately 20% of households were classified differently by method 5.

**Table 3:** Percentage agreement between different methods of identifying catastrophic health expenditure

	Total budget (10%)	Total budget (25%)	Actual food spending (40%)	Partially normative food (40%)	Normative food, rent, utilities (40%)
Total budget (10%)	–				
Total budget (25%)	88%	–			
Actual food spending (40%)	91%	97%	–		
Partially normative food (40%)	89%	98%	98%	–	
Normative food, rent, utilities (40%)	78%	78%	80%	82%	–

#### Catastrophic health expenditure by household budget

**Figure 3:**
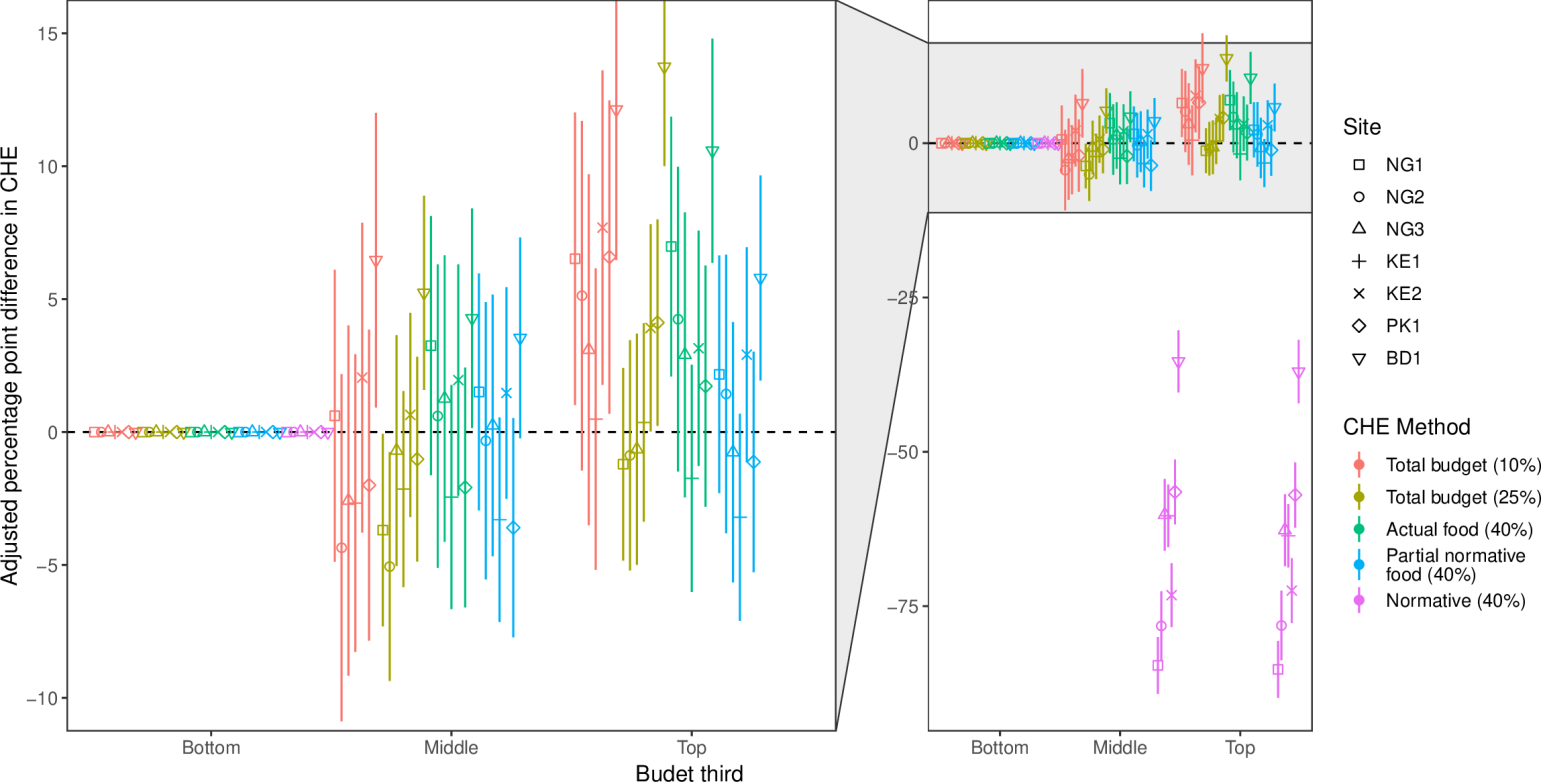
Adjusted percentage point difference in the proportion of the population experiencing catastrophic health expenditure between thirds of the distribution of household budget per equivalent person. The left-hand panel is a zoom in of the main plot on the right where indicated, with the results of the first four methods. BD1, Dhaka, Bangladesh; CHE, catastrophic health expenditure; KE1 and KE2, Nairobi, Kenya; NG1 and NG2, Ibadan, Nigeria; NG3, Lagos, Nigeria; PK1, Karachi, Pakistan.

Figure 3 shows the relationship between household budget and CHE. There was evidence that, for all but the normative method (method 5), better-off households were more likely to experience CHE particularly using method 1, although the differences were variable (−1 percentage points (pp) to 12 pp higher in top third vs bottom third) and not consistent between sites. According to the normative method, households in the top third were 60–80 pp less likely to experience CHE than those in the bottom third, suggesting the normative method identified worse-off households in particular.

## Discussion

### Economic variation in slums

These slums are relatively small communities with populations ranging between 5000 and 60 000 people. Yet there exists a wide distribution of household budgets within slum sites. The Gini coefficients for the sites ranged from 0.33 to 0.48 and the 90/10 ratio was 4–7 in five sites and over 30 in two Nigerian sites. For comparison, among the Organisation of Economic Cooperation and Development (OECD) countries, Gini coefficients for individual metropolitan areas have been estimated to range from approximately 0.20 to 0.45,^17^ and in the UK the 90/10 ratio of incomes was approximately 4.^18^ While comparable figures are not available for low and middle income country (LMIC) metropolitan areas, the results suggest that slums are not concentrated areas of extreme poverty within a city, but have a heterogeneous population with inequality comparable to that observed at even the level of whole countries.

### Financial circumstances and healthcare need and use

The inequality in household financial circumstances translates into inequities in healthcare access and use as worse-off households seek healthcare less frequently. Our results suggest that differences in use between individuals from better and worse-off households are explained by individuals as differences in their perceived need for healthcare rather than differences in their ability to access it or actual need for healthcare, particularly since in all our sites there were a wide range of public and private providers available within short distances.^19^ The differential recognition and assessment of health needs by income and education has been well documented both between and within countries^20 21^; here we show this likely extends even within small communities. Several mechanisms may be at play, including different expectations about health status, the role of education in the recognition of symptoms or the willingness to recognise symptoms when there are competing work needs.

Low healthcare use provides fewer opportunities to identify signs of non-communicable illness, such as cancers, which present in stage 3 or 4 despite the availability of diagnostic and therapeutic technology that could have identified and treated the cancer in earlier stages.^22 23^ We would hypothesise therefore that the inequalities in budget, and hence healthcare use, translate into inequalities in the prognosis of diseases like cancers even within small communities like slums.

### Should we target UHC initiatives at the slum-level?

In addition to differences in the recognition and reporting of symptoms, the propensity to seek care may also be lower among worse off households due to the relative costs of doing so. Across the African continent, in a large number of countries, survey evidence shows that households with greater wealth use healthcare services more frequently, but not because they are more likely than poorer people to benefit from accessing those services.^24 25^ Similar evidence has been shown for South Asian and Southeast Asian countries. Our results are focused at a much smaller scale, but they tell the same story.

The inequality of income and healthcare use in slums suggests that the ‘slum’ categorisation may be of limited value in directing UHC policy. Some authors have suggested that improved education among the poor may be a solution to these inequalities in use of health services to better enable people to perceive healthcare needs.^24 26^ However, even in our sites with reasonably homogeneous education levels, the same patterns were observed, and even after adjusting for education levels, individuals from better-off households were more likely to report experiencing a need for healthcare and consulting.

### Should we use CHE to monitor local UHC progress?

CHE is a widely used UHC indicator of health systems functioning designed to identify the incidence of financial hardship arising due to healthcare use. It was used in a recent global survey of ‘progress towards UHC’.^4^ However, our results echo those of Cylus *et al*,^8^ who showed that the most widely used methods of calculating CHE reflect lower barriers to use healthcare for better-off households rather than hardship or affordability.

Recent work has shown a global increase in the proportion of households spending over 10% and 25% of their budgets on healthcare,^11^ although it is not clear whether this is because of growth in global incomes or worsening financial protections and public support for healthcare (or both). One alternative measure we examined was a fully normative method that subtracted a context-specific amount needed for subsistence. The subsistence amount is based on an arbitrary cut-off in the distribution of spending in the community, but it discriminated between better-off and worse-off households with many households having zero or negative capacity to pay. However, as with all the methods, it is not clear if households identified as having CHE faced *unaffordable* spending. Households evidently made choices about their need for and use of healthcare based on their financial circumstances.

Households who face the sudden and devastating effects of diseases like tuberculosis or trauma will likely face unaffordable healthcare costs. CHE may be a useful measure of the distress caused by these particular circumstances. But as an aggregate measure of access to primary care, CHE is highly non-specific, variable and dependent on the methodology used, suggesting it is a poor measure for tracking UHC at local levels. CHE methods and their results are also affected by length of recall period of the questions and the level of disaggregation of spending in surveys,^27 28^ further raising questions about their validity.

### Strengths and limitations

We acknowledge several limitations to the result presented here. We did not assess the actual health status of the participants, so we cannot identify whether the differences in reported need and use reflect actual need. Given previous findings that indicate individuals who have a lower level of education or income are less likely to recognise illness and to report lower levels of severity,^20^ we have assumed that our results indicate inequitable differences in healthcare use rather than differences in health status. However, further research should address this topic. While every effort was made to ensure a representative sample, our response rates ranges from 70% to 95% across the study sites. Individuals in poorer health or from worse-off households may have been less likely to respond^29 30^, which may bias our results. However, we suggest that this may lead us to underestimate the degree of inequality in these communities.

## Conclusions

Our results suggest that the evaluation of any locally targeted policy to improve healthcare access and use, such as subsidies, clinical provision or otherwise, should consider the distribution and heterogeneity of effects across the targeted population and not just the mean effect. Some policies or interventions to improve access could result in widening inequalities and leave the very poorest behind. Strategies to improve use of health services will likely have to focus at very granular levels to be optimally effective since large-scale solutions, such as public funding of all healthcare free at the point of use, are likely infeasible for many LMICs presently. While some reports recognise the heterogeneity in slum populations, ‘slums’ are often treated in the academical and policy literature as an archetypal grouping of poor people.^5^ However, they are complex, heterogeneous areas with highly variable characteristics^31^ and it is unlikely that broad ‘slum-level’ policy solutions would be as successful as those that take a more granular view of the intended beneficiaries of these policies.

## Data Availability

Data are available upon reasonable request. The data are available from the study authors on request.
